# Postpartum Aortic-Origin Uterine Artery Pseudoaneurysm: Retroperitoneal Hematoma Treated With Coils

**DOI:** 10.7759/cureus.92944

**Published:** 2025-09-22

**Authors:** Kyle Johnson, Miles Howard, Shayan S Ahmad, Oliver A Kashyap

**Affiliations:** 1 Surgery, Arizona College of Osteopathic Medicine, Midwestern University, Glendale, USA; 2 Obstetrics and Gynaecology, Abrazo Community Health Network, Phoenix, USA; 3 Internal Medicine, Arizona College of Osteopathic Medicine, Midwestern University, Glendale, USA

**Keywords:** aberrant uterine artery, case report, coil embolization, interventional radiology, postpartum hemorrhage, uterine artery pseudoaneurysm, vascular anomaly

## Abstract

We report a rare case of massive left abdominopelvic hematoma (~16.6 × 13.9 × 14.5 cm) from a 1.3 cm pseudoaneurysm on an aberrant uterine artery from the anterior abdominal aorta, arising between the superior mesenteric artery and inferior mesenteric artery, supplying the lateral uterus in a woman in the postpartum period. The hematoma manifested on postpartum day two with signs of hypovolemia and a painful, throbbing lump in the left lower abdomen. Computed tomography (CT) angiography revealed a large retroperitoneal hematoma and a pseudoaneurysm associated with the anomalous artery, which also showed an aorta of normal caliber and no abdominal aortic aneurysm. Successful hemostasis was achieved using selective endovascular coil embolization with adjunct Gelfoam (Pfizer Inc., New York, United States). This case emphasizes vigilance for vascular anomalies in postpartum hemorrhage and demonstrates how advanced imaging and rapid endovascular intervention can prevent maternal morbidity and mortality.

## Introduction

Postpartum hemorrhage (PPH) remains one of the leading causes of maternal morbidity and mortality worldwide, accounting for up to 27% of maternal deaths globally [1]. Although most PPH cases are attributed to uterine atony, trauma, retained placental tissue, and coagulopathy, rare etiologies such as vascular anomalies should also be considered, particularly in atypical presentations or cases refractory to standard management [2]. Pseudoaneurysms and aberrant vascular anatomy are infrequent but potentially fatal sources of hemorrhage. Aberrant arterial branches from the abdominal aorta are exceedingly rare and typically asymptomatic; however, during pregnancy, hormonal and hemodynamic changes can render these vessels susceptible to pseudoaneurysm formation and rupture, especially in the postpartum period [3]. We present a case involving a rarely reported aberrant arterial branch from the abdominal aorta that enlarged in the retroperitoneal space and was successfully managed with endovascular embolization.

## Case presentation

A 32-year-old gravida 4, para 3 (G4P3) woman was admitted at 37.4 weeks gestation for induction of labor with a dichorionic diamniotic twin pregnancy. Her perinatal course was complicated by hyperemesis gravidarum, iron deficiency anemia, and rapid plasma reagin (RPR)-positive syphilis, all of which were appropriately treated. She underwent an uncomplicated spontaneous vaginal delivery, and her immediate postpartum course was unremarkable.

On postpartum day two, the patient developed sudden-onset, severe left lower abdominal pain associated with a painful, throbbing lump beneath the skin, hypotension (blood pressure 84/49 mmHg), tachycardia (heart rate 122 bpm), and pallor. On physical examination, she exhibited left flank tenderness without any vaginal bleeding. Her hemoglobin dropped from 11.2 to 7.8 g/dL. Laboratory results are summarized in Table 1.

**Table 1:** Laboratory results

Test	Patient value	Reference range
Hemoglobin	7.8 g/dL	12–16 g/dL
White blood cell count	8.5 ×10⁹/L	4–11 ×10⁹/L
Platelets	210 ×10⁹/L	150–400 ×10⁹/L
Aspartate aminotransferase (AST)	22 U/L	10–40 U/L
Alanine aminotransferase (ALT)	19 U/L	7–56 U/L
International normalized ratio (INR)	1.0	0.9–1.1

**Figure 1:**
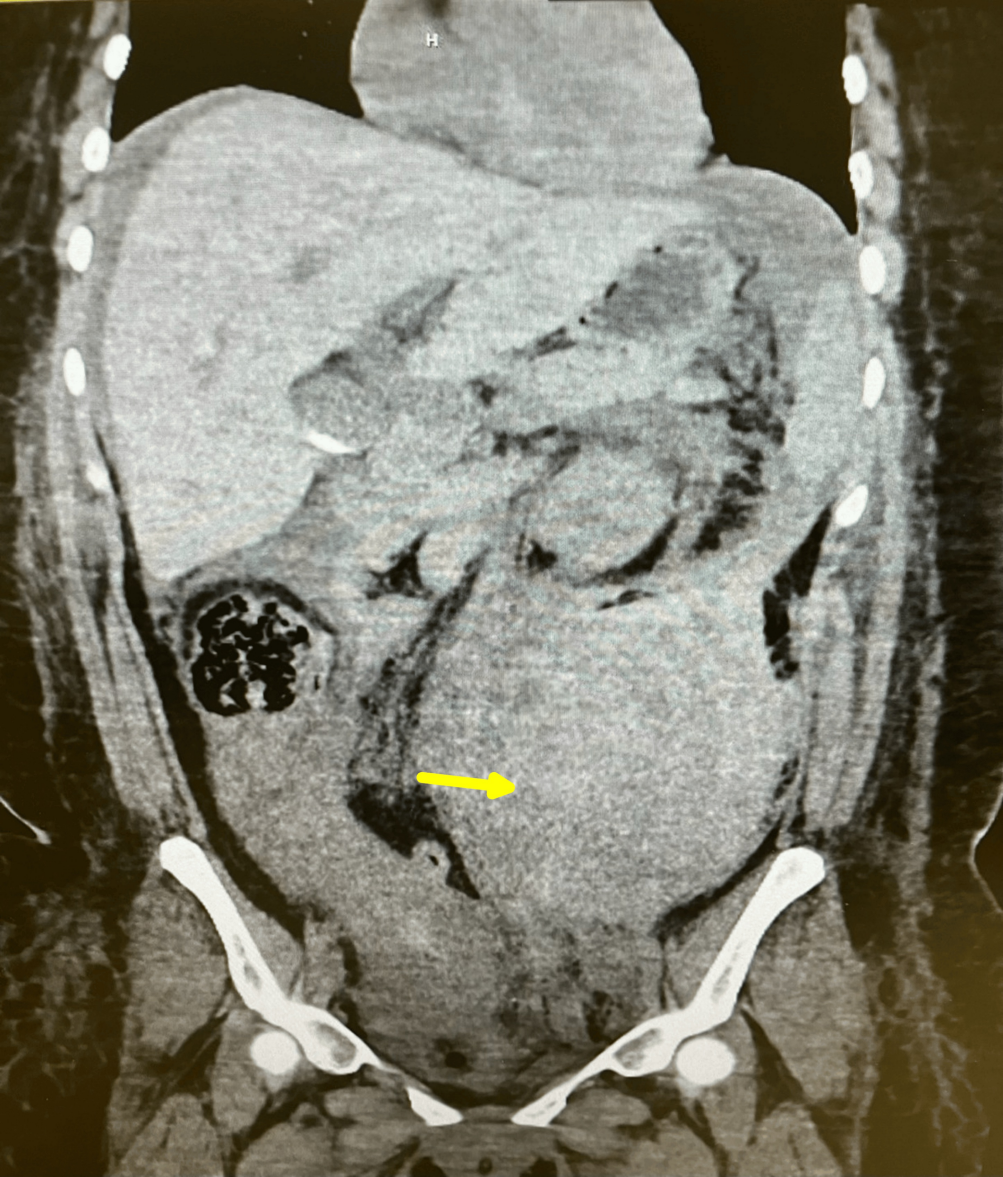
Coronal CT angiography showing a large retroperitoneal hematoma (arrow) measuring 16.6 x 13.9 x 14.5 cm with an adjacent pseudoaneurysm.

**Figure 2:**
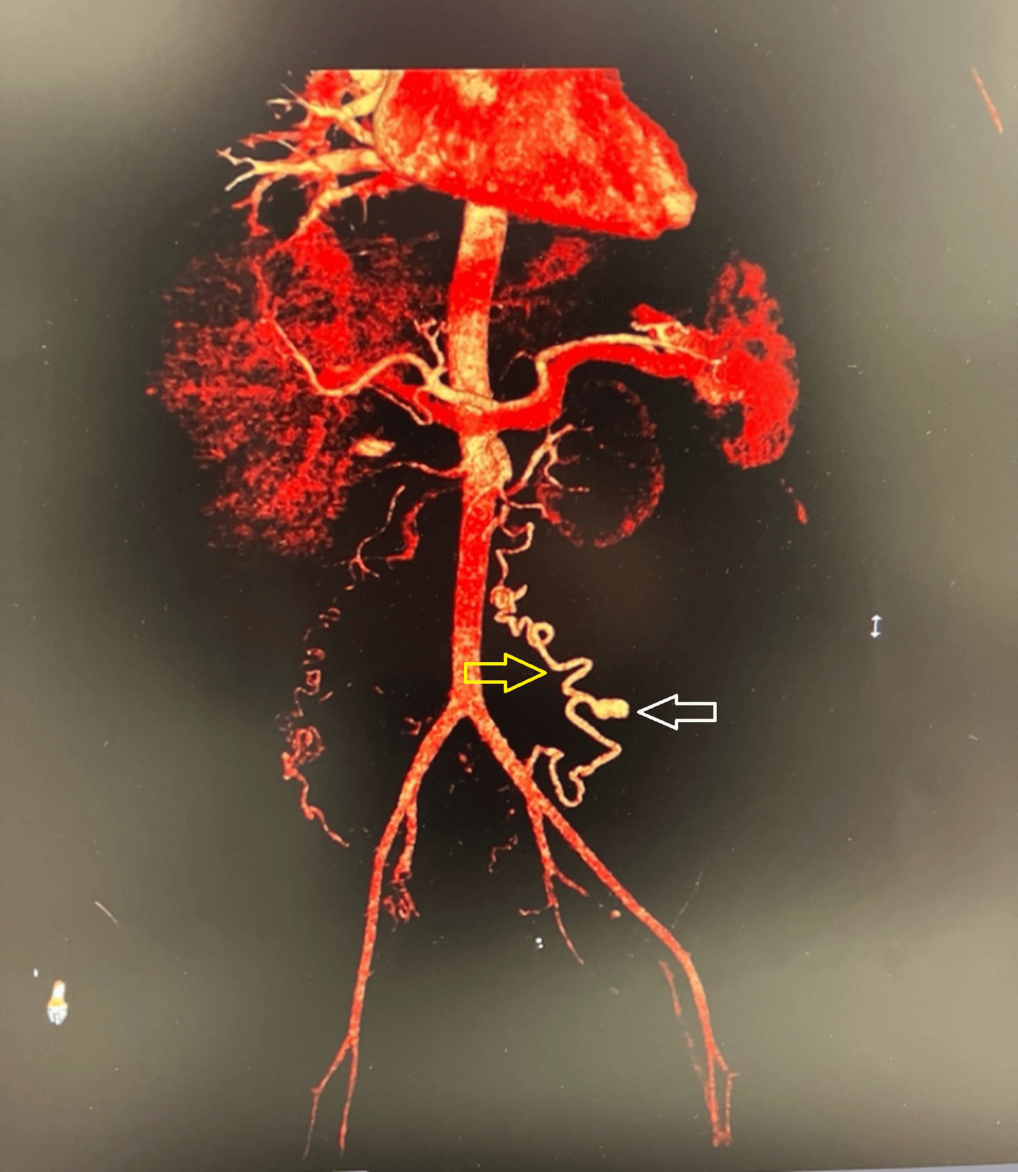
Arteriogram revealing an aberrant uterine artery originating directly from the abdominal aorta with contrast extravasation from the pseudoaneurysm. Yellow arrow indicates the artery and white arrow indicates the aneurysm

Computed tomography (CT) angiography of the abdomen and pelvis (Figure 1) revealed a massive retroperitoneal hematoma with active extravasation from a 1.3 cm pseudoaneurysm arising from a tortuous arterial branch originating directly from the anterior abdominal aorta at the L2-L3 vertebral level, believed to be an aberrant uterine artery, distinct from the normal uterine arterial anatomy. The patient was urgently taken to interventional radiology (Figure 2). Selective catheter angiography confirmed the presence of the aberrant uterine artery with active contrast extravasation.

**Figure 3:**
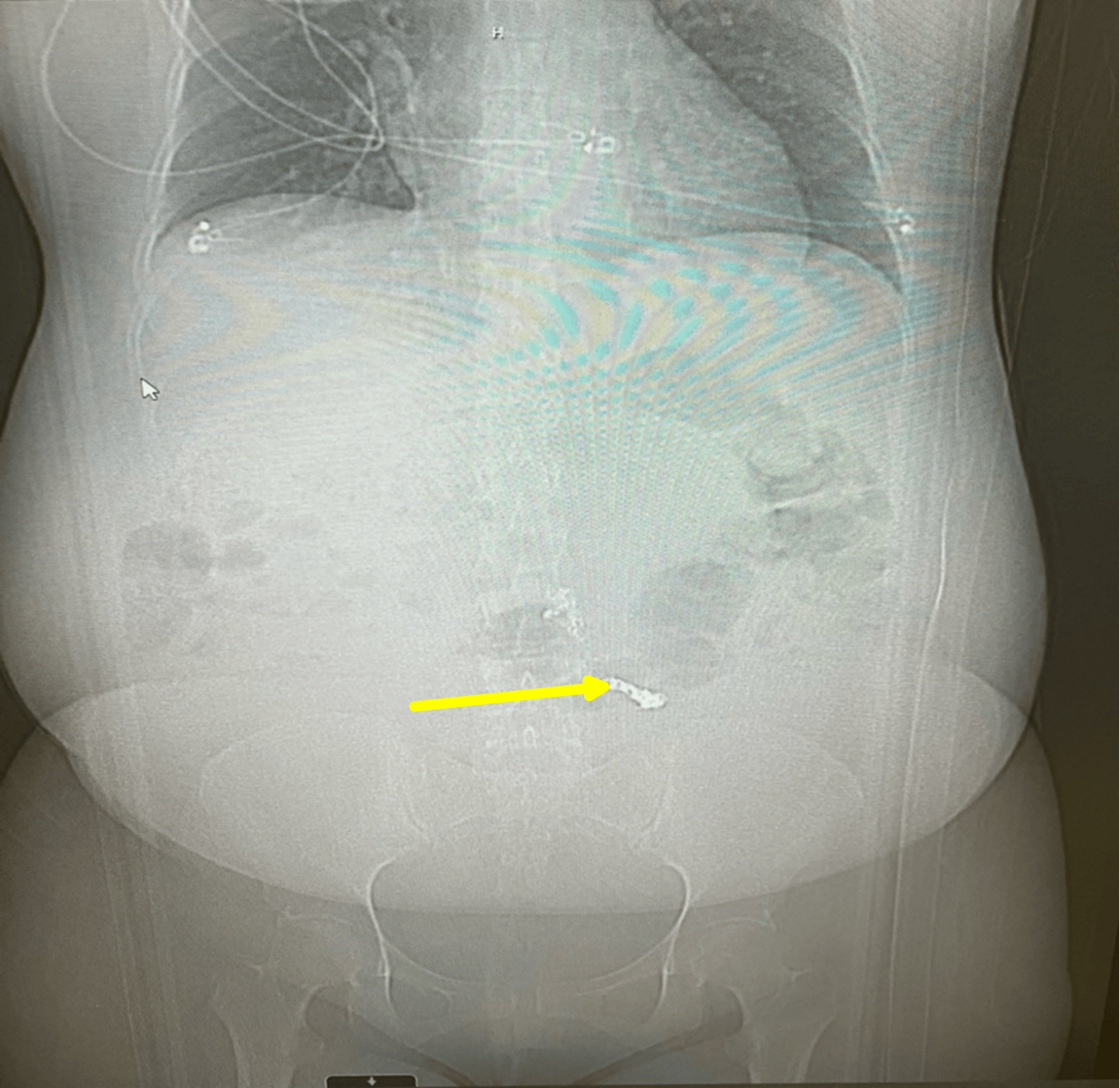
Superselective embolization using Ruby coils (Penumbra Inc.) and Gelfoam slurry (Pfizer Inc.), achieving complete occlusion. Abdominal X-ray demonstrates coil position in aberrant artery.

**Figure 4:**
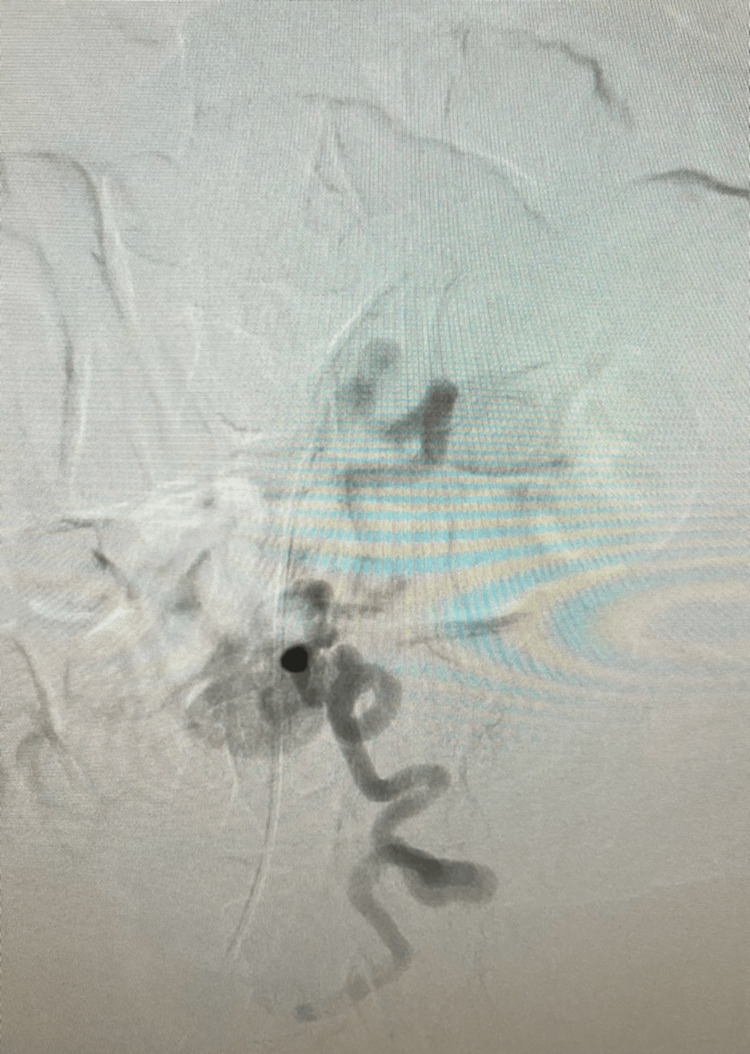
Pre-embolization fluoroscopic image showing active contrast extravasation from aberrant artery prior to coil deployment.

**Figure 5:**
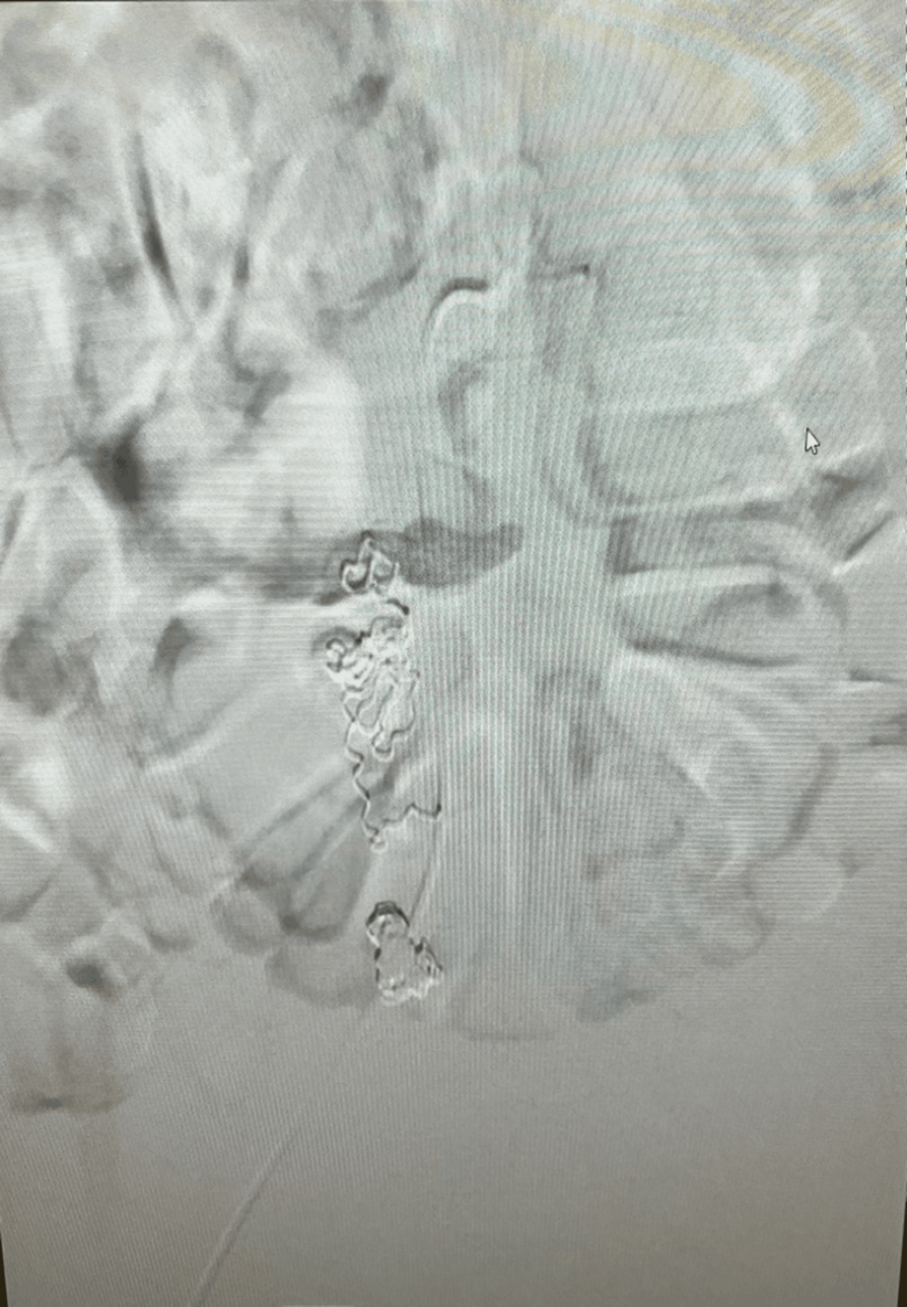
Post-embolization fluoroscopic image showing successful occlusion of the pseudoaneurysm with no further contrast leakage.

Superselective embolization (Figure 3) was performed using Ruby coils (Penumbra Inc., Alameda, California, United States) and Gelfoam slurry (Pfizer Inc., New York, United States), achieving complete occlusion and cessation of bleeding. Abdominal X-ray confirmed coil placement post-intervention. Pre-embolization fluoroscopic imaging (Figure 4) demonstrated active contrast extravasation from the aberrant artery prior to coil deployment, while post-embolization imaging (Figure 5) showed complete occlusion with no further contrast leakage.

Following the procedure, the patient was stabilized hemodynamically with four units of packed red blood cells (PRBC) and was monitored in the intensive care unit (ICU). Repeat CT angiography showed no further hematoma enlargement. She was discharged home in stable condition on postpartum day six with conservative management of the hematoma. The patient had no plans for future pregnancy and had inquired about a tubal ligation vs a vasectomy for her husband.

## Discussion

This case illustrates an exceptionally rare presentation of retroperitoneal hemorrhage from a pseudoaneurysm on an aberrant uterine artery arising directly off the abdominal aorta between the superior mesenteric artery and inferior mesenteric artery during the postpartum period. Normally, uterine arteries arise from the anterior division of the internal iliac arteries and course medially to supply the uterus. Variants of uterine and ovarian arterial supply have been described, including rare origins and collateral pathways, and recognition of such variants is critical for targeted therapy [4-7]. Rarely, uterine arterial supply has been reported in proximity to the aortic origin of the ovarian artery, underscoring the need to search for nonstandard inflow when bleeding persists [6]. Aberrant arteries can arise from persistence of primitive anastomoses or abnormal regression during embryologic development [8]. Although usually asymptomatic, such vessels may become hemodynamically significant in pregnancy due to increased flow and hormonal effects on the vascular wall, predisposing to pseudoaneurysm formation and rupture [3,6,9,10].

The retroperitoneal location of bleeding without vaginal hemorrhage complicated recognition, an important reminder that postpartum hypotension, flank or abdominal pain, and acute anemia warrant prompt cross-sectional imaging to localize atypical sources. In this case, contrast-enhanced CT angiography identified both the active extravasation and the unusual vascular origin, enabling rapid, targeted endovascular therapy by Interventional Radiology (IR). Selective arterial embolization achieves high primary success for obstetric hemorrhage (>90%) with lower morbidity than surgical options, though outcomes are influenced by etiology (e.g., abnormal placentation) and coagulopathy [11]. Multidisciplinary coordination among obstetrics, radiology, and IR teams was essential to achieving hemostasis while preserving uterine function.

This case is notable for three features. First, the culprit vessel arose directly from the anterior abdominal aorta at the L2-L3 level, a rare site relevant to procedural planning. Second, hemorrhage presented as a retroperitoneal hematoma without vaginal bleeding, reinforcing the need for high clinical suspicion in atypical PPH. Third, minimally invasive super-selective embolization achieved durable hemostasis as documented on post-procedural imaging.

## Conclusions

Aberrant arteries, although rare, should be considered in atypical postpartum presentations, particularly when classical signs (e.g., vaginal bleeding) are absent. Early CT angiography to define vascular anatomy and prompt IR-guided embolization can be lifesaving. Awareness of vascular variants and interdisciplinary collaboration are critical to the emergent management of complex PPH.

## References

[1] Say L, Chou D, Gemmill A (2014). Global causes of maternal death: a WHO systematic analysis. Lancet Glob Health.

[2] Rath WH (2011). Postpartum hemorrhage--update on problems of definitions and diagnosis. Acta Obstet Gynecol Scand.

[3] Pelage JP, Soyer P, Repiquet D (1999). Secondary postpartum hemorrhage: treatment with selective arterial embolization. Radiology.

[4] Liapis K, Tasis N, Tsouknidas I, Tsakotos G, Skandalakis P, Vlasis K, Filippou D (2020). Anatomic variations of the uterine artery: review of the literature and their clinical significance. Turk J Obstet Gynecol.

[5] Yi SW (2017). Extravasating uterine pseudoaneurysm: a rare cause of postpartum haemorrhage. J Obstet Gynaecol.

[6] Sugai S, Nonaka T, Tamegai K, Sato T, Haino K, Enomoto T, Nishijima K (2021). Successful repeated uterine artery embolization in postpartum hemorrhage with disseminated intravascular coagulation: a case report and literature review. BMC Pregnancy Childbirth.

[7] Arfi A, Arfi-Rouche J, Barrau V, Nyangoh Timoh K, Touboul C (2018). Three-dimensional computed tomography angiography reconstruction of the origin of the uterine artery and its clinical significance. Surg Radiol Anat.

[8] Moore KL, Persaud TVN, Torchia MG (2016). The Developing Human: Clinically Oriented Embryology. https://search.worldcat.org/title/900261075.

[9] Oyelese Y, Ananth CV (2010). Postpartum hemorrhage: epidemiology, risk factors, and causes. Clin Obstet Gynecol.

[10] Abdul-Kadir R, McLintock C, Ducloy AS (2014). Evaluation and management of postpartum hemorrhage: consensus from an international expert panel. Transfusion.

[11] Descargues G, Douvrin F, Degré S (2001). Abnormal placentation and selective embolization of the uterine arteries. Eur J Obstet Gynecol Reprod Biol.

